# Polymer Nanocomposite Based on Pyrolyzed Polyacrylonitrile Doped with Carbon Nanotubes: Synthesis, Properties, and Mechanism of Formation

**DOI:** 10.3390/polym16101308

**Published:** 2024-05-07

**Authors:** Irina Zaporotskova, Olesya Kakorina, Lev Kozhitov, Dmitriy Muratov, Natalia Boroznina, Sergei Boroznin, Alexandra Panchenko

**Affiliations:** 1 Institute of Priority Technologies, Volgograd State University, Universitetskii Prospect, 100, Volgograd 400062, Russia; olessya.08@mail.ru (O.K.); boroznina.natalya@volsu.ru (N.B.); boroznin@volsu.ru (S.B.); alexandra1889@mail.ru (A.P.); 2 Institute of New Materials, National Research Technological University “MISIS”, Leninsky Prospekt, 4, Moscow 119049, Russia; kozitov@rambler.ru (L.K.); muratovdg@yandex.ru (D.M.); 3 Institute of Petrochemical Synthesis A.V. Topchiev Russian Academy of Sciences, Leninsky Prospekt, 29, Moscow 119991, Russia

**Keywords:** polyacrylonitrile (PAN), carbon nanotubes, filler, modification, quantum chemical calculations, carbon-polymer nanocomposites, reflection coefficient, absorption coefficient, adsorption complex, pyrolyzed polyacrylonitrile

## Abstract

The paper investigates the possibility of fabricating a carbon nanotubes (CNT)-modified nanocomposite based on pyrolyzed polyacrylonitrile (PPAN). The layered structure of PPAN ensures the attachment of nanotubes (NT) to the polymer matrix, forming enhanced PPAN/CNT nanocomposites. We synthesized a PPAN/CNT polymer nanocomposite and investigated its mechanical, conductive, and electronic properties. Using the quantum chemical method density functional theory (DFT), we studied an interaction mechanism between PPAN and single-walled carbon nanotubes. We described the structural features and electron energy structure of the obtained systems. We found that the attachment of a CNT to the PPAN matrix increases tensile strength, electrical conductivity, and thermal stability in the complex. The obtained materials were exposed to electromagnetic radiation and the dielectric constant, reflection, transmission, and absorption coefficients were measured. The study demonstrates the possibility of using carbon nanotubes for reinforcing polyacrylonitrile polymer matrix, which can result in the development of an enhanced class of materials possessing the properties of both polymers and CNTs.

## 1. Introduction

Modern society uses polymers in various industries, including electronics, medicine, and construction. However, there is a growing demand for materials with improved properties as the existing ones do not always meet the performance requirements. To meet challenges, researchers are developing methods of fabricating polymer nanostructures with enhanced properties by modifying known materials with additives [1,2,3,4,5]. CNTs are promising candidates for reinforcing various polymers as they have remarkable mechanical strength, rigidity, and electrical conductivity. Incorporated into polymers, CNTs transfer their properties to the fabricated materials, yielding durable and lightweight nanocomposites [6,7,8,9].

Earlier research [10] showed that nanotubes introduced to the polymeric matrix enhanced its load-bearing capacity and mechanical properties and the nanocomposites obtained demonstrated excellent tensile strength, toughness, and wear resistance compared to pure polymers. Moreover, the high electrical conductivity of carbon nanotubes makes it possible to develop conductive polymers, which is promising for electronics [11,12,13].

Previous studies demonstrated that nanotubes improved the mechanical properties of polymer panels, reducing their weight [6] and increasing the efficiency of products based on epoxy resins [7]. In experimentation and theoretical research [8], the authors described carbon fiber plastic modification with carbon nanotubes. Paper [9] was devoted to enhancing the strength properties of polymers by testing the wear resistance of a sponge structure comprised epoxy resin and carbon nanotubes. A three-dimensional CNT sponge was prepared and incorporated into the epoxy through the monomer infusion process. The fracture toughness and critical energy release rate of the nanocomposites were enhanced by more than 100% and 250%, respectively. The toughening mechanism behind such observation was revealed by studying the in situ crack initiation and propagation in the bulky nanocomposites with the assistance of a micro-mechanical testing device in a scanning electronic microscope chamber. The results showed that the intrinsic fracture behaviors, such as void generation, as well as the extrinsic toughening features arising from crack deflection and bifurcation, were responsible for the improved fracture properties of nanocomposites.

Carbon nanotubes also find their use in additive technologies as they improve the characteristics of the polymers taken as a base, as shown in [10]. This study examined the result of the introduction of Polybutylene Terephthalate (PBT) to acrylonitrile butadiene styrene (ABS) and explored the effects of the incorporation of Multi-Walled Carbon Nanotubes (MWCNT) to an ABS/PBT blend with different filler fractions (0.1, 0.3, and 0.5 wt.%) on the mechanical properties of the printed specimens. In order to assess the mechanical behavior of the printed parts, a standard melt flow index (MFI) and tensile, three-point flexural, and notched impact tests have been performed on the produced parts. The morphological analyses through Scanning Electron Microscopy (SEM) micrographs have been conducted to inspect the distribution of impregnated MWCNTs and the fracture behavior of the specimens. The most satisfactory improvement has been observed in printed parts of the ABS/PBT/CNT nanocomposites containing 0.3 wt.% of MWCNTs. By integrating CNTs into biocompatible polymers, the researchers have developed materials with exceptional properties, including enhanced mechanical strength and electrical conductivity [7,14]. These advances promote the creation of next-generation implants, tissue engineering frameworks, and drug delivery systems. Moreover, the ability of carbon nanotubes to facilitate electrical stimulation of cells is promising for applications in neurology, regenerative medicine, and biosensors.

The rapid development of nano- and optoelectronics a d nanophotonic devices with increased power capacity [12] requires developing enhanced absorbing materials for protection against electromagnetic radiation. The next generation of absorbing materials will allow reducing mutual interference between devices operating in the radio and near-infrared ranges [12]. We see that there is an urgency for theoretical and practical studies of the basics of synthesis and physical properties of improved nanocomposite nanomaterials that can provide high efficiency of absorption or reflection of electromagnetic radiation in the barrier band of the device [15,16,17,18,19,20].

To date, nanocomposites based on a carbon-containing polymer matrix receive the focus of attention. Polymers have low thermal conductivity but the remarkable thermal conductivity of carbon nanotubes that ensures efficient heat transfer inside the polymer matrix can reduce this limitation. The described property is of primary importance in electronic devices, where effective temperature control is crucial, as it affects the performance and durability of the device [13,14,21]. 

The well-known polymer polyacrylonitrile (PAN), characterized by an ordered structure and composition, which depend on the synthesis methods [22,23], is one of the efficient and promising materials used as a matrix for CNTs introduction. With 68% of carbon, PAN demonstrates low weight loss during synthesis due to thermal transformations. When exposed to IR heating, polyacrylonitrile undergoes significant chemical and structural changes. First, heating contributes to the emergence of a developed polyconjected system and next, PAN molecules crosslink to form a layered structure (PPAN). Heated above 600 °C, it transforms into graphite-like carbon. The layered structure of the transition form of PAN between polymer and carbon can exhibit semiconductor properties [23,24]. The advantages of PPAN include low cost, simplicity of synthesis, and the possibility of controlled pyrolysis, which allows materials with specified parameters and new functional characteristics to be obtained [19,20]. Pyrolyzed polyacrylonitrile can serve as a matrix for creating nanocomposites with nanoparticles of metals or alloys [22].

The paper investigates the possibility of fabricating a polymer nanocomposite based on pyrolyzed polyacrylonitrile modified with carbon nanotubes. The structure of PAN will contribute to the “entanglement” of nanotubes in the polymer matrix and the formation of an enhanced PAN/CNT nanocomposite. We synthesized the polymer nanocomposite PAN/CNT and studied its mechanical and conductive properties. Using the quantum chemical DFT (density functional theory) method, we investigated an interaction mechanism between PPAN and single-walled carbon nanotubes and modeled the structural features and electron energy structure of the obtained polymer nanocomposite. Also, the relevance of this work is determined by the fact that such theoretical and practical studies were conducted for the first time.

## 2. Experimental Section

### 2.1. Materials

The study used OcSiAl’s TUBALL single-walled carbon nanotube (SWCNT) (CAS: 308068-56-6) (purity: >75%, average diameter: 1.6 ± 0.4 nm, length: >5 microns). Polyacrylonitrile (PAN) with a molecular weight of 100–150 thousand units was synthesized in the presence of a redox catalytic system [23,24], namely dimethylformamide (DMFA) (chemically pure) (Acros Organics, Antwerpen, Belgium).

### 2.2. The Technique of Synthesizing Nanocomposite PAN/CNT Films

Several types of nanocomposite films of polyacrylonitrile filled with single-walled carbon nanotubes (SWCNTs) with different concentrations of CNTs (from 0 to 30 wt.%) were synthesized.

Nanocomposite films were obtained in several stages:

1—preparation of a 5 wt.% solution of PPAN in a dimethylformamide (DMFA) solvent;

2—ultrasonication for 30 min to obtain a suspension of SWCNT in DMFA;

3—ultrasonication of PPAN solution and SWCNT suspension for 1 h;

4—drying the nanocomposite film in a vacuum drying cabinet at a temperature of 40 °C for 6 h, followed by holding at rt for 3 days.

As a result, depending on the CNT content and the amount of DMFA solvent, films with a thickness of 40 to 100 microns were obtained.

We dissolved PAN (0.25 g) in 5 mL of DMFA for 6 h at 50–60 °C to obtain a 5% solution of PAN-DMFA. Furthermore, to obtain the dispersion state for carbon nanotubes to introduce them into the PAN solution, we carried out several steps. Liquid mixtures were prepared, including the DFMA solution of various volumes and carbon nanotubes taken in various proportions to obtain the required concentrations (from 0 to 30 mass%). Depending on the concentration required to produce PAN/SWCNT nanocomposites, the volume of DMFA into which nanotubes were injected varied from 5 mL to 0.5 mass.% up to 50 mL for 30 mas.%. Then, we placed the prepared liquid mixtures of SWCNT+DFMA in the ultrasonic dispersant MEF93.T, where they were exposed to the ultrasonic treatment of 250 W/cm^2^ at a frequency of 22 kGC for 2 h. Furthermore, a solution of PAN-DMFA was added to the SWCNT-DMFA suspension with subsequent ultrasound treatment in the dispersant of MEF93.T for an hour, which ensured a uniform distribution of components in the mixture. It is important that in this way, stable suspensions were obtained, as after two months of control observation, we found no sediment in the suspensions, regardless of the concentrations of SWCNTs.

Then, we applied the PAN-SWCNT-DMFA suspension to the glass substrates of a rectangular shape of a size of 100 × 30 × 3 mm. The thickness of the films ranged from 40 to 100 microns due to different amounts of solvent (DMFA) at various SWCNT concentrations. Therefore, to obtain films of equal thickness, the suspension of PAN-SWCNT-DMFA was applied in several layers with subsequent drying to evaporate the solvent.

**Figure 1:**
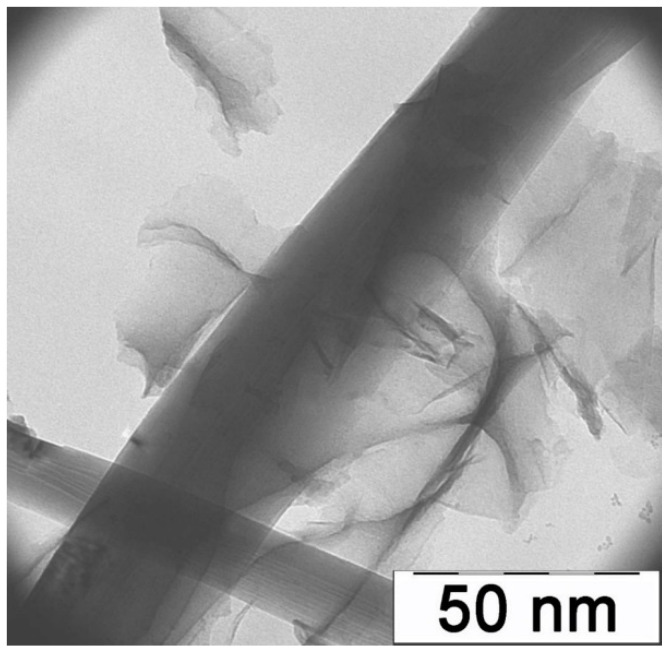
Micrography of the PAN/SWCNT nanocomposite.

We examined the resulting samples using a LEO912 AB OMEGA transmission electron microscope, accelerating voltage 60–120 kV, and magnification 80×–500,000×. The micrograph of PAN/SWCNT nanocomposite films at the magnification of 30,000× (Figure 1) clearly shows an isolated carbon nanotube surrounded by films of pyrolyzed polyacrylonitrile, which proves the high degree of the dispersion state of the system with introduced carbon nanotubes.

We carried out mechanical tests to determine the breaking load of films on the M350-5 AT Testometric testing machine. The test speed was 1 mm/min. At least three samples of films with the same concentration of SWCNT were tested.

The resistivity of the samples was measured using the Cryotel device of the same name. A 4-probe method with a linear arrangement of probes was used. Measurements were made at several points and averaged. No special materials were used to create contacts.

The electromagnetic properties of nanocomposites were studied in the frequency range from 130–250 GHz using the submillimeter spectrometer IMZ TD-01. The spectrometer IMZ TD-01. A reverse wave lamp (RWL) was used as a radiation source. The measurements were carried out according to the method described in [25].

**Figure 2:**
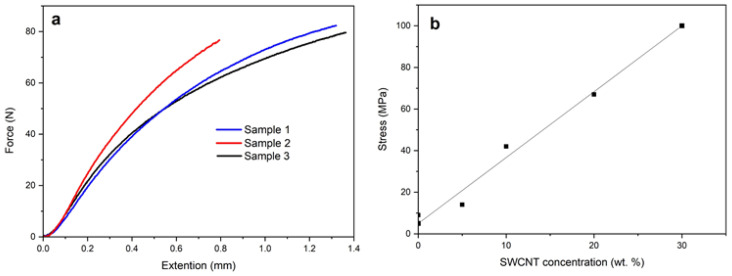
The results of mechanical tests of the PAN/SWCNT film (30 wt.% CNT) (**a**) and the dependency of the breaking load of PAN/CNT films and the concentration of carbon nanotubes (**b**).

Mechanical tests showed the impact of the SWCNT concentration on the strength of the obtained films. The analysis of the obtained results showed that as the concentration of SWCNT in solution increases from 0 to 30 wt.%, the breaking load of the nanocomposite film increases from 9 to 100 MPa and the dependence is almost linear (Figure 2). Apparently, the percolation threshold is already reached at low concentrations.

**Figure 3:**
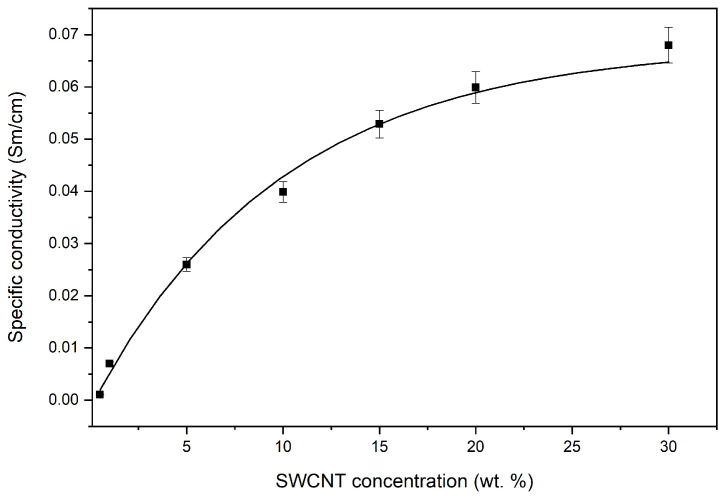
The dependence of the specific electrical conductivity of nanocomposites on the concentration of SWCNTs.

**Figure 4:**
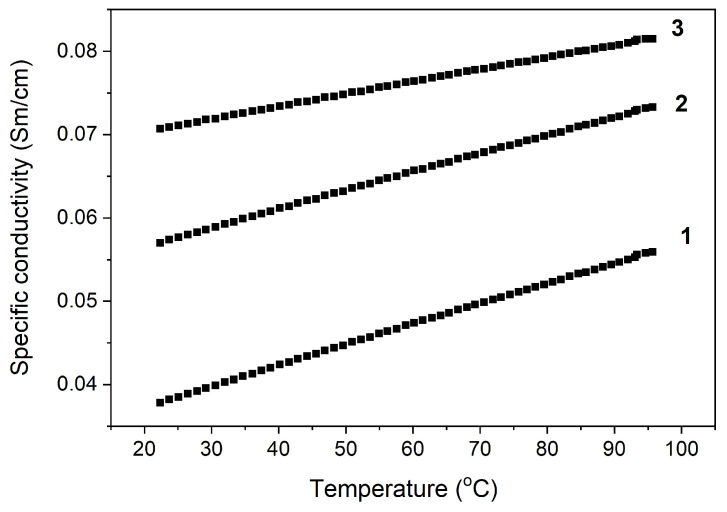
Temperature dependence of the specific electrical conductivity of PAN nanocomposites/SWCNT: 1—10 wt.%, 2—20 wt.%, and 3—30 wt.%.

The results of the study of the effect of the SWCNT content on the electrical conductivity of nanocomposites at room temperature are shown in Figure 3. It was established that an introduction of 0.5 wt.% SWCNT causes a change in electrical conductivity. With a higher concentration of SWCNT, electrical conductivity did not increase; at 10 wt.%, electrical conductivity increased fourfold and in the concentration range of 10–30 wt.%, the electrical conductivity increased twofold, which is typical for materials with percolation mechanisms of electrical conductivity. We can conclude that the electrical conductivity of the PAN/CNTs nanocomposites is determined by the degree of percolation, which depends on the CNT’s length, concentration, and orientation in the polymer matrix. A gradual increase in the electrical conductivity of nanocomposites at higher temperatures was also revealed (Figure 4).

**Figure 5:**
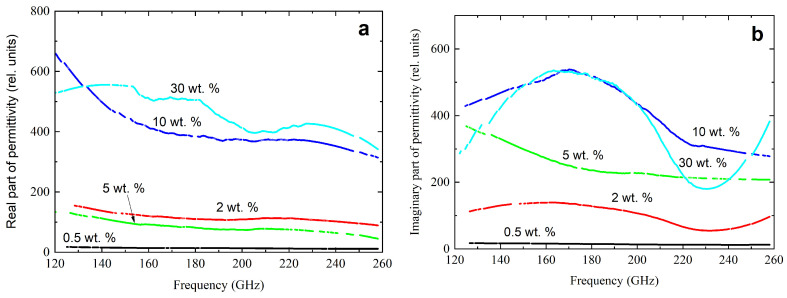
Frequency dependencies of the real (**a**) and imaginary part (**b**) of the complex dielectric constant in the frequency range from 120 Hz to 260 GHz for nanocomposites with different CNT content.

A submillimeter spectrometer in the frequency range of 120–260 GHz was used to measure the complex dielectric permittivity of nanocomposites. The results are presented in Figure 5.

With a higher frequency, a decrease in the actual part of the complex dielectric constant was observed. An increase in the concentration of CNTs caused a sharp increase in the values of the real and imaginary parts of the complex dielectric constant.

**Figure 6:**
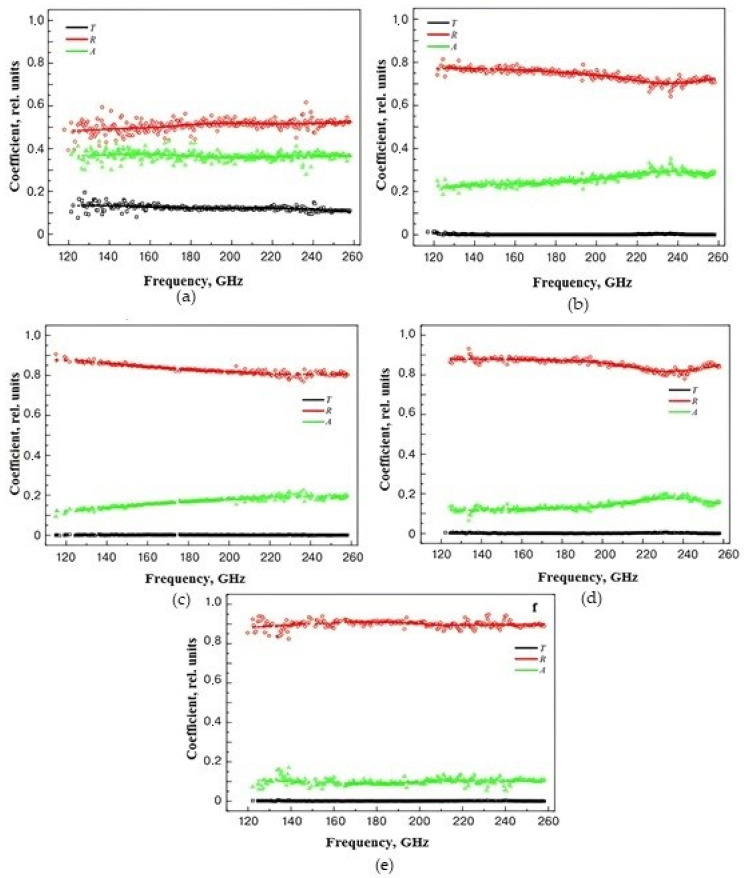
Frequency dependence of reflection coefficient (R), transmission coefficient (T), and absorption coefficient (A) for samples of nanocomposite materials with CNT concentrations: (**a**)—0.5 wt.%, (**b**)—2 wt.%, (**c**)—5 wt.%, (**d**)—10 wt.%, and (**e**)—30 wt.%.

In order to evaluate the interaction of materials with electromagnetic radiation, the absorption properties were calculated. The results obtained are shown in Figure 6. An increase in the concentration of CNTs leads to an increase in the reflection coefficient and a decrease in the level of transmitted power. Starting with samples with a CNT concentration of 5 wt.%, the absorption coefficient level was in the range of 10–20%. The loss level for samples with a concentration of 0.5 wt.% was significantly higher than for other structures, most likely due to its polarization properties.

## 3. The Computer Modeling of the PPAN/SWCNT Interaction

The mechanisms of interaction between pyrolyzed polyacrylonitrile and single-walled carbon nanotubes, which lead to the formation of a polymer nanocomposite PPAN/SWCNT, were investigated. Calculations were performed within the framework of the molecular cluster model using the DFT method with the Becker-Lee-Yang-Parr (B3LYP) function and the 6–31G basis.

The DFT method with the B3LYP functional is based on a combination of three components: Lee-Yang-Parr function for exchange energy, the Becker-Lee-Yang-Parr function for correlation energy, and the Gilles-Johansen function for exchange–correlation energy. This combination provides a balance between accuracy and computational efficiency. B3LYP has good accuracy for calculating bond energies and geometric parameters [26].

In the DFT method, the central physical quantity is the electron density p, which depends on the coordinates of all the electrons that form the system. The electron density created by all the electrons of a molecule is determined by

(1) $$\rho \left(r\right)={\sum_{i=1}^{N}|\phi_{i}\left(r\right)|}^{2}$$

$\phi_{i}$—electron wave function.

In the theory of the Kohn—Sham density functional, the total energy of the system is expressed as the function of the charge density.

(2) $$E[n]=\langle \Psi \left[n\right]|\hat{T}+\hat{U}+\hat{V_{ext}}|\Psi \left[n\right]\rangle =T+U+V_{ext}=\;T_{S}+{vV}_{H}+V_{ext}+\left(T-T_{S}+U+V_{H}\right)$$

T—kinetic energy of interacting particles, $T_{S}$—kinetic energy of a free particles, $V_{ext}$—exchange–correlation energy, U—the energy of the Coulomb interaction, and $V_{H}$—Hartree energy.

DFT methods differ from each other in the form of the E_XC_(p) functional, as well as in the presence of various fitting parameters.

Correlation exchange functional of B3LYP:

(3) $$E_{XC}^{B3LYP}=(1-\alpha )E_{X}^{LSDA}+aE_{X}^{HF}+b{\Delta E}_{X}^{B88}+(1-c)E_{C}^{VWN}+c{\Delta E}_{C}^{LYP}$$

$E_{X}^{LSDA}$—exchange energy using the local spin density approximation; $E_{X}^{HF}$—Hartree-Fock exchange energy; ${\Delta E}_{X}^{B88}$—three-parameter Becke functional of the gradient correction method; $E_{C}^{VWN}$—approximation of the local spin density VWN to the correlation functional; ${\Delta E}_{C}^{LYP}$—correlation functional proposed by Lee, Yang and Parr; and a, b, and c are constants selected from experimental data for relatively simple chemical compounds: a = 0.2, b = 0.72, and c = 0.81.

**Figure 7:**
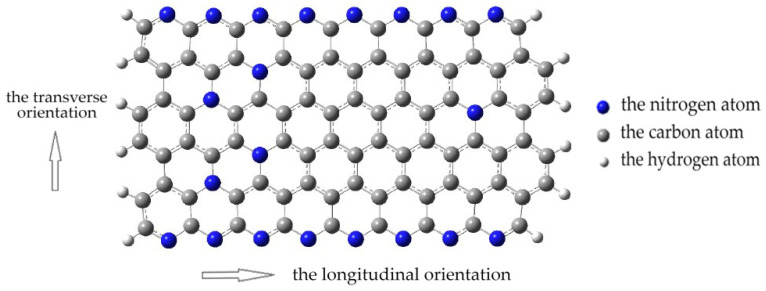
A molecular cluster of pyrolyzed polyacrylonitrile: the blue beads indicate nitrogen atoms, the gray beads indicate carbon atoms, and the white beads indicate hydrogen atoms. The arrows show carbon nanotubes orientation—the transverse orientation to the monolayer of the PPAN cluster or the longitudinal orientation to the monolayer of the PPAN cluster.

Let us consider the model used for quantum chemical calculations of the interaction between a monolayer of pyrolyzed polyacrylonitrile and single-walled carbon nanotubes. The PPAN monolayer fragment consisted of 118 atoms, the percentage of which was as follows: 72% were carbon atoms, 17.8% were nitrogen atoms, and 10.2% were hydrogen atoms. A carbon nanotube chosen was an armchair CNT type with chirality (3.3). The nanotube molecular cluster contained 60 carbon atoms. Figure 7 shows a fragment of a PPAN monolayer with arrows indicating the longitudinal and transverse directions used in further model experiments.

**Figure 8:**
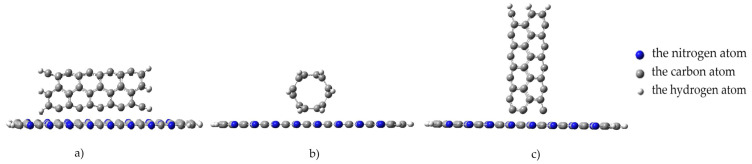
Orientation types of a nanotube to a monolayer of pyrolyzed polyacrylonitrile: (**a**) parallel–longitudinal orientation to the PPAN monolayer; (**b**) parallel–transverse orientation to the PPAN monolayer; and (**c**) perpendicular orientation to the PPAN monolayer.

To simulate an interaction process, we modeled a nanotube movement toward the monolayer with an increment of 0.1 Å along an axis drawn perpendicular to the plane of the monolayer. We studied several positions for the nanotube orientation to the surface of the monolayer to consider all possible cases of interaction between the nanostructures under study. The position where a carbon nanotube was oriented parallel to the monolayer, its axis parallel to the longitudinal arrow in Figure 7 (Figure 8a). The position where a nanotube was rotated at an angle of 90 degrees relative to its previous position, while maintaining an orientation parallel to the monolayer. In this position, the nanotube axis is oriented parallel to the transverse arrow in Figure 7 (Figure 8b). The position where a nanotube was oriented perpendicular to the monolayer and its axis coincided with the perpendicular drawn to the monolayer surface along which the nanostructures approached each other (Figure 8c).

**Figure 9:**
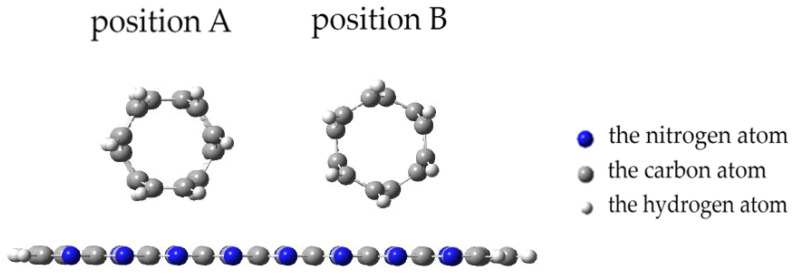
Position A and Position B of a CNT parallel orientation to the PPAN monolayer.

When a nanotube is oriented parallel to the surface of the monolayer, there are two options for a CNT approaching the PPAN monolayer surface. Figure 9 shows two positions: Position A and Position B. In Position A, the nanotube face formed by hexagons is parallel to the monolayer and the carbon atoms at the vertices of the hexagons are at an equal distance from the monolayer. In position B, the nanotube rotates; its side face, consisting of hexagons, turns at an angle of 120 degrees to the monolayer and only 10 atoms out of 60 forming the side vertices of these hexagons along the nanotube are at the closest distance to the PPAN monolayer.

**Figure 10:**
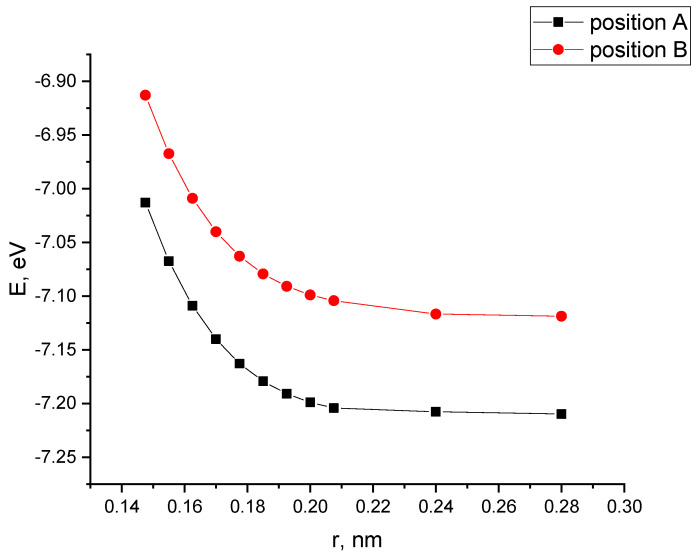
The potential energy curves of the interaction between CNTs and a PAN monolayer with a parallel-longitudinal orientation method for the two tube Positions A and B, E−interaction energy, and r-a distance between a carbon nanotube and the PPAN layer.

The performed calculations of the interaction of CNTs with the PAN monolayer for the case when a nanotube is oriented parallel to the longitudinal axis of the PAN monolayer allowed us to construct profiles of the potential energy of interaction for two positions A and B (see Figure 9), shown in Figure 10. The analysis of the curves found that when a nanotube is oriented parallel to the longitudinal axis of the PPAN layer, the creation of a stable complex is impossible as the curve showed no energy minimum.

**Figure 11:**
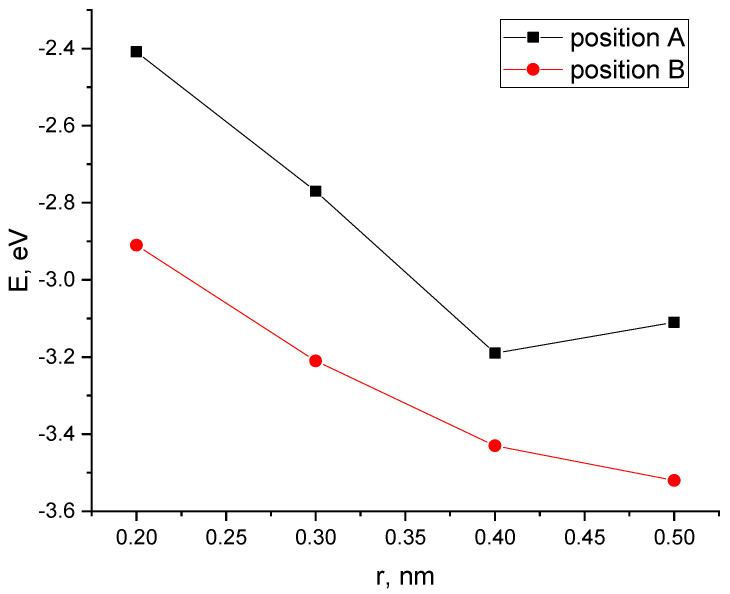
The potential energy curves of the interaction between CNTs and a PAN monolayer with a parallel-transverse orientation method for the two tube positions: Position A and Position B, E—interaction energy, and r-a distance between a carbon nanotube and the PPAN layer.

Next, Position A and Position B were considered with a nanotube oriented parallel to the transverse axis of the PPAN monolayer. A CNT was moving to the surface incrementally, as a result of which energy curves of this process were constructed (Figure 11).

The analysis of the results established the possibility of the existence of the complex “PPAN+CNT” for Position A (a CNT is oriented to the monolayer with a side face when the carbon hexagons are parallel to the PPAN plane): there is a minimum on the curve corresponding to a distance of 0.4 nm and the interaction energy is 3.19 eV.

**Figure 12:**
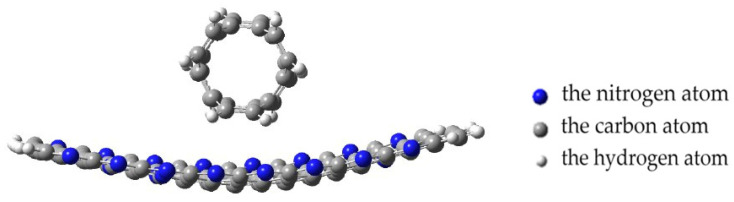
Changing the geometry of the complex with a parallel–transverse arrangement of CNTs to the PAN layer, position A; the blue beads indicate nitrogen atoms, the gray beads indicate carbon atoms, and the white beads indicate hydrogen atoms.

The analysis of the geometry of the systems showed that the approach of a nanotube to the PPAN monolayer causes a change in the planarity of the layer. The layer in the area of the nanotube attachment is deformed (bent) and the nanotube turns toward the localization of nitrogen atoms in the pyrolyzed polyacrylonitrile layer until it occupies a stable position. The rotation was 7 degrees relative to the original location of the CNT (Figure 12).

For the parallel–transverse orientation of a CNT in Position B (a CNT’s carbon atoms located at the vertices of the hexagons a CNT are oriented parallel to the PPAN monolayer), there is no minimum on the curves, which indicates the impossibility of interaction between the CNT and the PPAN layer in this arrangement.

**Figure 13:**
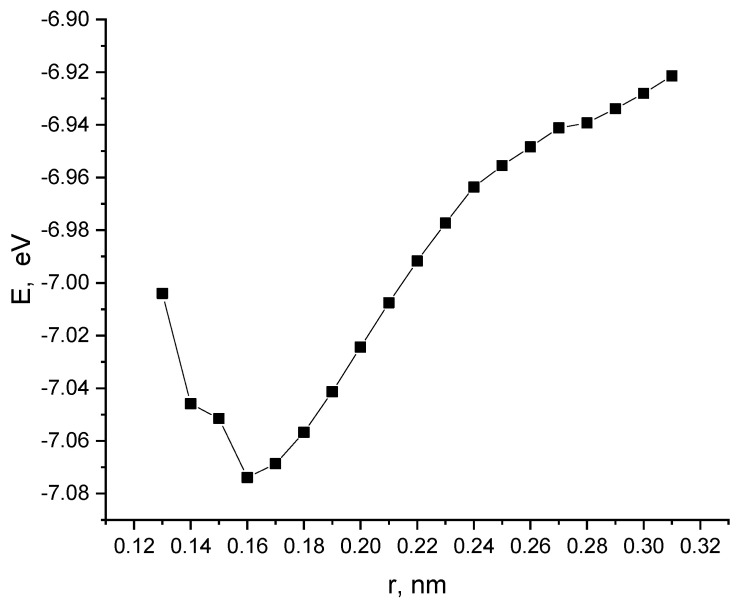
The potential energy curve of the interaction between a CNT and a PPAN layer when a CNT is oriented perpendicular to the monolayer of PPAN.

Next, the interaction of a nanotube with a monolayer of pyrolyzed polyacrylonitrile was modeled with a perpendicular orientation of the nanotube to the layer. The calculations performed made it possible to construct a profile of the potential energy of the process (Figure 13). Analysis of the obtained values of the interaction distance r, corresponding to the minimum on the energy curve, established the fact of chemical adsorption of CNTs on the surface of the layer (r = 0.16 nm and E = 7.07 эB).

**Figure 14:**
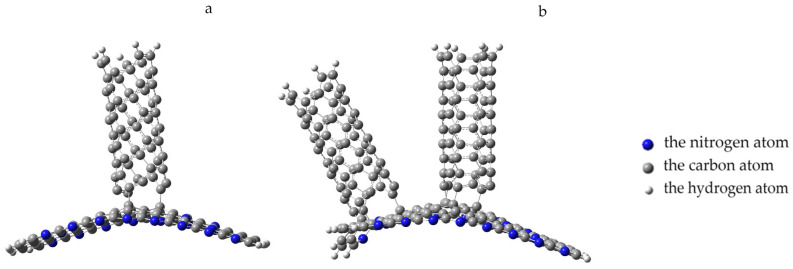
Formation of an adsorption complex: (**a**) “PPAN+CNT” and (**b**) “PPAN+2CNT”; the blue beads indicate nitrogen atoms, the gray beads represent carbon atoms, and the white beads indicate hydrogen atoms.

The analysis of the geometry of an interaction between the polymer and nanotube showed the following. The monolayer of PPAN began to bend toward the nanotube at a distance of 0.35 nm between them. After that, a chemical bond between the carbon atoms of the nanolayer and the nanotube was formed when the distance decreased to 0.16 nm. The final stage was the inclination of the nanotube by 10 degrees to the nanolayer relative to the main axis and the formation of another bond between carbon atoms. Thus, three chemical bonds C-C were formed between the polymer atom and the tube; the average bond length is 0.16 nm and the interaction energy is −7.07 eV (Figure 14a).

Next, the attachment of a nanotube (3.3) to the adsorption complex “PPAN+CNT” was considered. The nanotube was oriented parallel to the tube that had been adsorbed onto the polymer monolayer, the distance between the nanotubes was 0.25 nm and the average distance to the polymer monolayer was 0.45 nm. The process was modeled by the increment approach of another nanotube to the PPAN monolayer surface. The analysis of the geometric parameters of the process aimed at searching for a stable position of the nanotube relative to the “PPAN+CNT complex”, which showed the following. The nanotube approached the polymer layer by 0.05 nm, after which further approximation was accompanied by a shift of the CNT relative to the initial position, the angle of deviation was 45 degrees; at a distance of 0.2 nm, the polymer atoms began to interact with the boundary atoms of the CNT, namely, they shifted toward it. At a distance of 0.17 nm, four C-C bonds were formed between the nanotube and polymer atoms; then, a decrease in the bond length was observed to a value of 0.16 nm and an interaction energy value was 9.53 eV (Figure 14b).

The theoretical studies made it possible to obtain the electronic energy and geometric characteristics of polymer nanocomposites with CNTs, presented in Table 1. The binding energies of the resulting nanocomposites were calculated for different orientations of a single-walled carbon nanotube to the surface of a PPAN monolayer. The binding energy values, comparable to the energy values of stable pure PPAN, proved that the resulting PPAN+CNT nanocomposite complexes are stable.

**Figure 15:**
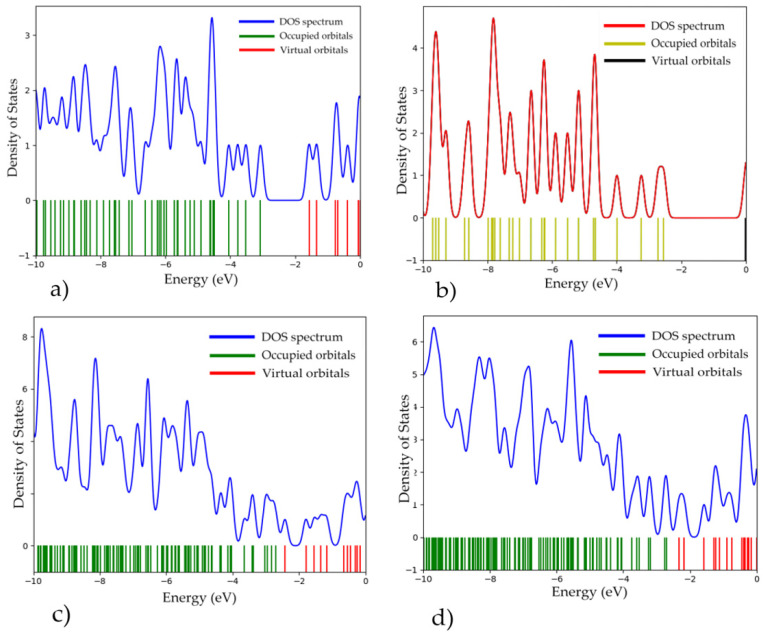
Single-electron spectra and density of states (DOS): (**a**) PPAN; (**b**) CNT(3.3); (**c**) “PPAN+CNT”—physical adsorption (position A with a parallel–transverse arrangement); and (**d**) “PPAN+CNT”—chemical adsorption (a perpendicular arrangement).

Single-electron spectra of the obtained complexes were constructed (Figure 15) and the band gap ΔEg of the systems was determined. It was found that the interaction of CNTs with a polymer resulted in a narrower band gap width compared with the width of pure PPAN (Table 1). The electron energy and geometric characteristics of the polymer nanocomposites with CNTs are presented in Table 1.

So, the completed studies have proven the possibility of creating “PPAN+CNT” complexes, leading to the production of a polymer nanocomposite

## 4. Conclusions

A method for producing SWCNT/PPAN nanocomposites was developed, which allowed us to synthesize nanocomposite films containing from 0.5 to 30 wt.% of single-walled carbon nanotubes. It was found that the use of fillers in the form of SWCNT in a polymer nanocomposite based on PPAN significantly affected the mechanical properties of the obtained polymer. The research results showed that the thermal stability of the samples increased with a higher concentration of SWCNT in the nanocomposite. With the introduction of SWCNT, nanocomposites displayed an enhanced electrical conductivity of 0.5 to 30 wt.%, compared to pure PPAN.

The study of the electromagnetic characteristics of PPAN/SWCNT nanocomposites showed that the reflection coefficient depends non-linearly on the concentration of carbon nanotubes and the minimum reflection coefficient was observed at a concentration of 0.5 wt.%. Materials with an SWCNT concentration of more than 5 wt.% showed a nearly identical reflection coefficient at a sufficiently low transmission coefficient, which is obviously due to the percolation effects of conductivity at high frequencies as well as the polarization properties of nanocomposites.

Quantum chemical studies of interaction mechanisms between the nanocomposite components showed that a PPAN-based doping with single-walled carbon nanotubes stable polymer nanocomposite was formed due to the sorption interaction between a CNT and a PPAN monolayer during the synthesis of a nanocomposite polymer material ‘PPAN/SWCNT’. One of the crucial conditions was the orientation of the nanotube to the surface of the PPAN layer. The SWCNT formed stable complexes when it was oriented perpendicular to the PPAN surface. The interaction of CNTs with the monolayer caused the curvature of the initially planar PPAN layer while the structure retained its stability. The PPAN/SWCNT complex had a narrower band gap than the monolayer of pyrolyzed polyacrylonitrile, allowing control of its conductive properties. The result was consistent with the obtained experimental results.

## Figures and Tables

**Table 1:** Characteristics of polymer nanocomposites with CNTs: E_bn_—binding energy, ∆E—band gap width, E_ad_—adsorption energy, and R_ad_—average adsorption distance.

Structure	E_bn_, eV	∆E_g,_ eV	E_ad_, eV	R_ad_, nm
PPAN	9.03	1.19		
CNT (3.3)	8.81	2.54		
“PPAN+CNT” (position A with parallel–transverse arrangement)	8.98	0.28	−3.19	0.44
“PPAN+CNT” (perpendicular arrangement)	8.43	0.37	−7.07	0.16
“PPAN+2CNT” (perpendicular arrangement)	8.35	1.05	−9.53	0.16

## Data Availability

Data are contained within the article.
